# The effects of neoadjuvant chemoradiotherapy and an in-hospital exercise training programme on physical fitness and quality of life in locally advanced rectal cancer patients (The EMPOWER Trial): study protocol for a randomised controlled trial

**DOI:** 10.1186/s13063-015-1149-4

**Published:** 2016-01-13

**Authors:** Lisa Loughney, Malcolm A. West, Graham J. Kemp, Harry B. Rossiter, Shaunna M. Burke, Trevor Cox, Christopher P. Barben, Michael G. Mythen, Peter Calverley, Daniel H. Palmer, Michael P. W. Grocott, Sandy Jack

**Affiliations:** Anaesthesia and Critical Care Research Area, NIHR Respiratory Biomedical Research Unit, University Hospital Southampton NHS Foundation Trust, CE93, MP24, Tremona Road, Southampton, SO16 6YD UK; Integrative Physiology and Critical Illness Group, Clinical and Experimental Sciences, Faculty of Medicine, University of Southampton, CE93, MP24, Tremona Road, Southampton, SO16 6YD UK; Department of Musculoskeletal Biology and MRC – Arthritis Research UK Centre for Integrated research into Musculoskeletal Ageing (CIMA), Faculty of Health and Life Sciences, University of Liverpool, Liverpool, UK; Academic Unit of Cancer Sciences, Faculty of Medicine, University of Southampton, South Academic Block, Tremona Road, Southampton, SO16 6YD UK; School of Biomedical Sciences, Faculty of Biological Sciences, University of Leeds, Clarendon Way, Leeds, LS2 9JT UK; Rehabilitation Clinical Trials Center, Division of Respiratory and Critical Care Physiology and Medicine, Los Angles Biomedical Research Institute, 1124 W Carson St, Torrance, CA 90502 USA; Departments of Molecular and Clinical Cancer Medicine and Biostatistics, University of Liverpool, Liverpool, UK; Colorectal Surgery Research Group, Aintree University Hospitals NHS Foundation Trust, Liverpool, UK; Anaesthesia and Critical Care, University College London, London, UK; Institute of Ageing and Chronic Disease, Aintree University Hospital, Liverpool, UK; Department of Molecular and Clinical Cancer Medicine, Institute of Translational Medicine, University of Liverpool, UK and The Clatterbridge Cancer Centre NHS Foundation Trust, Wirral, UK

**Keywords:** Neoadjuvant chemoradiotherapy, Exercise training, Physical activity, Surgery, Surgical outcome

## Abstract

**Background:**

The standard treatment pathway for locally advanced rectal cancer is neoadjuvant chemoradiotherapy (CRT) followed by surgery. Neoadjuvant CRT has been shown to decrease physical fitness, and this decrease is associated with increased post-operative morbidity. Exercise training can stimulate skeletal muscle adaptations such as increased mitochondrial content and improved oxygen uptake capacity, both of which are contributors to physical fitness. The aims of the EMPOWER trial are to assess the effects of neoadjuvant CRT and an in-hospital exercise training programme on physical fitness, health-related quality of life (HRQoL), and physical activity levels, as well as post-operative morbidity and cancer staging.

**Methods/Design:**

The EMPOWER Trial is a randomised controlled trial with a planned recruitment of 46 patients with locally advanced rectal cancer and who are undergoing neoadjuvant CRT and surgery. Following completion of the neoadjuvant CRT (week 0) prior to surgery, patients are randomised to an in-hospital exercise training programme (aerobic interval training for 6 to 9 weeks) or a usual care control group (usual care and no formal exercise training). The primary endpoint is oxygen uptake at lactate threshold ($\overset{\cdotp }{\mathrm{V}}{\mathrm{o}}_2$ at ${\widehat{\uptheta}}_{\mathrm{L}}$) measured using cardiopulmonary exercise testing assessed over several time points throughout the study. Secondary endpoints include HRQoL, assessed using semi-structured interviews and questionnaires, and physical activity levels assessed using activity monitors. Exploratory endpoints include post-operative morbidity, assessed using the Post-Operative Morbidity Survey (POMS), and cancer staging, assessed by using magnetic resonance tumour regression grading.

**Discussion:**

The EMPOWER trial is the first randomised controlled trial comparing an in-hospital exercise training group with a usual care control group in patients with locally advanced rectal cancer. This trial will allow us to determine whether exercise training following neoadjuvant CRT can improve physical fitness and activity levels, as well as other important clinical outcome measures such as HRQoL and post-operative morbidity. These results will aid the design of a large, multi-centre trial to determine whether an increase in physical fitness improves clinically relevant post-operative outcomes.

**Trial registration:**

ClinicalTrials.gov NCT01914068 (received: 7 June 2013). Sponsor: University Hospital Southampton NHS Foundation Trust.

## Background

The standard treatment pathway for magnetic resonance (MR)-defined, locally advanced rectal cancer is neoadjuvant chemoradiotherapy (CRT) followed by total mesorectal excision surgery [1, 2]. Chemotherapy, combined with radiotherapy, improves local disease control and local recurrence for locally advanced rectal cancer [3–5]. With optimised local treatment, including neoadjuvant CRT and surgery, local relapse rates have now been reduced to less than 10 % [2]. Cancer is associated with cachexia, which, in the pre-operative period, has been shown to influence perioperative outcome, increasing the risk of complications, mortality and length of hospital stay in major gastrointestinal surgery [6]. Chemotherapy has been related to skeletal muscle wasting, oxidative stress, mitochondrial death [7] and reduced *in vivo* mitochondrial function [8]. Furthermore, cancer treatment has been linked to decreased physical fitness levels, which appear to be related to the type of treatment; that is, physical fitness is lower in those receiving surgery and radiotherapy in combination with chemotherapy than in those who receive radiotherapy or surgery alone [9]. Neoadjuvant CRT has been shown to be associated with a decrease in objectively measured physical fitness, as measured by cardiopulmonary exercise testing (CPET) in those with locally advanced rectal cancer [10]. This decrease in physical fitness was in turn shown to be associated with increased short-term post-operative morbidity [10]. Moreover, a decrease in physical fitness has also been shown in a group of upper gastrointestinal cancer patients following neoadjuvant chemotherapy [11]. In this case, the change was associated with 1-year mortality [11].

CPET is a good pre-operative risk assessment tool as it provides an objective global assessment of the integrative responses of the pulmonary, cardiovascular and musculoskeletal systems rather than evaluating the function of individual organ systems in isolation [12]. Furthermore, as a dynamic assessment, CPET provides more insight than a resting test into the response to physiological stress. The perioperative period is a time of physiological stress as the surgical stress response increases the metabolic rate, and consequently, tissue oxygen demand rises. By detecting abnormal exercise capacity and, consequently, reduced physiological reserve, CPET identifies patients at increased risk of complications and mortality. CPET is increasingly being used preoperatively to identify patients at high risk of perioperative morbidity and mortality [13–15].

Exercise training can stimulate skeletal muscle adaptations such as increased mitochondrial content and improved oxygen uptake capacity [16], both of which are contributors to physical fitness and could possibly reduce the adverse effects of cancer treatment. In 2013, Jones and Aflano [17] reported a series of observational studies that suggest higher levels of exercise may be associated with an improved prognosis in patients with solid tumours. Physical fitness is a modifiable prognosticator, and women with breast cancer who exercise at moderate intensity, 30 minutes or more per day on 5 days or more per week have been reported to have a lower risk of death [18]. Furthermore, women who were physically active following diagnosis of non-metastatic colorectal cancer have been shown to have a significantly lower risk of colorectal cancer–specific death or death from any cause [19]. Our group (Fit-4-Surgery) has previously shown in a non-randomised study that a 6-week moderate to severe intensity, interval exercise training programme, following neoadjuvant CRT and prior to surgery in patients with locally advanced rectal cancer resulted in a clinically relevant increase in physical fitness [20] and HRQoL [21].

In this manuscript, we describe the design of a parallel group randomised controlled multi-centre trial comparing an in-hospital exercise training programme to a usual care control group in patients undergoing neoadjuvant CRT and elective surgery for locally advanced rectal cancer. To the best of our knowledge, the EMPOWER trial is the first RCT of such an intervention in a neoadjuvant setting.

### Aims

The aims of this study are to evaluate the following hypotheses:

### Primary hypothesis

A 9-week, in-hospital, exercise training programme compared with a usual care control group (usual care; no formal exercise training) will result in a clinically significant difference in oxygen uptake at lactate threshold ($\overset{\cdotp }{\mathrm{V}}{\mathrm{o}}_2$ at ${\widehat{\uptheta}}_{\mathrm{L}}$; 2.0 mL.kg^-1.^min^−1^) assessed using CPET, following neoadjuvant CRT prior to elective cancer surgery.

### Secondary hypothesis

- An in-hospital exercise training programme compared with a usual care control group (usual care; no formal exercise training) will result in an improvement in psychological health benefits as assessed using semi-structured interviews and questionnaires such as the EORTC QLQ-30 and EQ-5D following neoadjuvant CRT prior to cancer surgery.
- Neoadjuvant CRT will result in a reduction in $\overset{\cdotp }{\mathrm{V}}{\mathrm{o}}_2$ at ${\widehat{\uptheta}}_{\mathrm{L}}$ as assessed using CPET.

### Exploratory hypotheses

A 9-week in-hospital exercise training programme compared with a usual care control group (usual care; no formal exercise training) will be associated with 1) greater physical activity, assessed using SenseWear accelerometer, following neoadjuvant CRT prior to cancer surgery; 2) lower day 5 surgical morbidity (using POMS); and 3) improved Magnetic Resonance (MR)-defined local rectal cancer staging assessed using the TNM classification system (tumour, nodes, metastasis) and tumour regression grading (mrTRG).

## Methods/Design

### Study design

The EMPOWER Trial is a parallel group randomised controlled trial in locally advanced rectal cancer patients undergoing both neoadjuvant CRT and elective surgery. The trial is funded by the National Institute for Health Research - Research for Patient Benefit Programme (PB-PG-0711-25093), approved by North West Centre for Research Ethics Committees (13/NW/0259) and registered with clinicaltrials.gov (NCT01914068).

### Recruiting hospitals

Five NHS hospitals are currently recruiting to the trial: the University Hospital Southampton (UHS) NHS Foundation Trust, Aintree University Hospitals NHS Foundation Trust, Royal Hampshire County Hospital, South Tees Hospitals NHS Foundation Trust and Royal Bournemouth Christchurch Hospitals.

### Participants

**Fig. 1:**
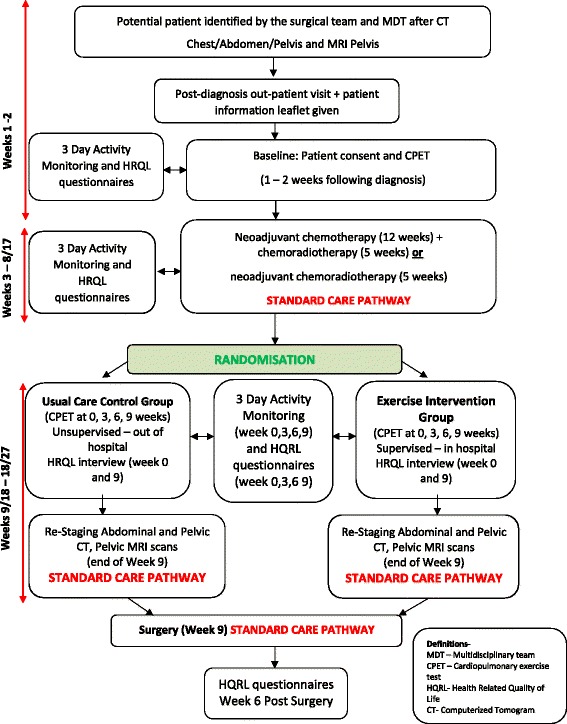
The EMPOWER trial algorithm presented illustrates the patient pathway and the time points of assessments and intervention as part of the trial

Eligibility criteria for inclusion at cancer diagnosis include the following: age ≥ 18 years, with MR-defined, locally advanced (circumferential resection margin threatened), resectable rectal cancer (≥ T2N + M0), undergoing standardised neoadjuvant CRT; with no distant radiological defined metastasis. Exclusion criteria include the following: inability to give informed consent, non-resectable disease, distant metastasis, inability to perform CPET or bicycle exercise, and patients who declined surgery or neoadjuvant CRT or who received non-standard neoadjuvant CRT. Figure 1 provides an algorithm of the clinical pathway and the complete series of assessments for the duration of the trial.

### Recruitment and randomisation

The EMPOWER trial is currently recruiting (start date is August 2013; end date is December 2015). All potentially eligible patients are being identified at multi-disciplinary meetings and approached with written information about the trial at the oncology/surgical outpatient appointment. Patients are contacted by telephone to provide additional information about the trial and to confirm their eligibility. If the patient chooses to participate in this trial, the first research visit is organised where written informed consent is obtained and all baseline measurements are undertaken. Following neoadjuvant CRT (week 0), patients are allocated to the in-hospital exercise training group or usual care control group. Patients are randomised (1:1) to either an in-hospital exercise training programme or usual care control group using the Trans European Network for patient randomisation in clinical trials system (TENAELA System).

### Interventions

#### Usual care control group

The usual care control group (no formal exercise training) receive routine care throughout their cancer pathway from diagnosis to surgical resection. No specific advice about exercise training is offered.

#### Exercise intervention group

The exercise training programme was designed to improve physical fitness in the time interval between end of neoadjuvant CRT and surgery. Exercise training begins on the first week following completion of neoadjuvant CRT, and the patients exercise in pairs for camaraderie. The exercise training programme is described below.

#### Exercise training protocol

The delivery of the exercise training programme is described using the FITT principle (frequency, intensity, time and type of exercise training (American College of Sports Science, 2009)) detailed below.

#### Exercise training frequency

Patients are requested to attend three in-hospital exercise training sessions per week for 6 to 9 weeks (dependent on the time interval between neoadjuvant CRT and surgery at each hospital).

#### Exercise training intensity

The exercise training is an aerobic interval exercise training programme incorporating moderate and severe intensities. Exercise training intensities are derived from each individual CPET derived at Week 0 (immediately post-CRT). Moderate-intensity exercise is at a power output equivalent to 80 % of the oxygen uptake at lactate threshold ($\overset{\cdotp }{\mathrm{V}}{\mathrm{o}}_2$ at ${\widehat{\uptheta}}_{\mathrm{L}}$). Severe-intensity exercise is at a power output half-way between $\overset{\cdotp }{\mathrm{V}}{\mathrm{o}}_2$ at ${\widehat{\uptheta}}_{\mathrm{L}}$ and $\overset{\cdotp }{\mathrm{V}}{\mathrm{o}}_2$ at peak (termed 50%Δ). Algebraically, this is calculated as follows:

Moderate-intensity exercise: (Work load at $$ \overset{\cdotp }{\mathrm{V}}{\mathrm{o}}_2 $$ at $$ {\widehat{\uptheta}}_{\mathrm{L}} $$ −$$ \raisebox{1ex}{$2$}\!\left/ \!\raisebox{-1ex}{$3$}\right. $$ of work ramp) × 80 %

Severe-intensity exercise: ((Work load at $$ \overset{\cdotp }{\mathrm{V}}{\mathrm{o}}_2 $$ at Peak-Work load at $$ \overset{\cdotp }{\mathrm{V}}{\mathrm{o}}_2 $$ at $$ {\widehat{\uptheta}}_{\mathrm{L}} $$ −$$ \raisebox{1ex}{$2$}\!\left/ \!\raisebox{-1ex}{$3$}\right. $$ of work ramp) × 50 %) + Work load at $$ \overset{\cdotp }{\mathrm{V}}{\mathrm{o}}_2 $$ at $$ {\widehat{\uptheta}}_{\mathrm{L}} $$.

Each exercise session included a 5-minute warm-up and cool-down using unloaded pedalling. Exercise training intensities are responsive to each CPET (informed by measured work rates at $\overset{\cdotp }{\mathrm{V}}{\mathrm{o}}_2$ at ${\widehat{\uptheta}}_{\mathrm{L}}$ and $\overset{\cdotp }{\mathrm{V}}{\mathrm{o}}_2$ Peak at Weeks 3 and 6) during the exercise programme and are derived and reported by two assessors. The absolute power output for subsequent training sessions are adjusted according to the outcome of the CPET.

#### Exercise training time

The first two training sessions involve 30 minutes of exercise which increases to 40 minutes per session thereafter. In the first week of training, patients perform the interval exercise training protocol for 20 minutes with a 5-minute warm-up and cool down. The interval exercise training phase includes four repeated bouts of moderate to severe intensity. Following week 1, the time of each exercise training session increases to 30 minutes with a 5-minute warm-up and cool down. The interval exercise training phase includes six repeated bouts of moderate- to severe-intensity intervals.

#### Exercise training type

**Fig. 2:**
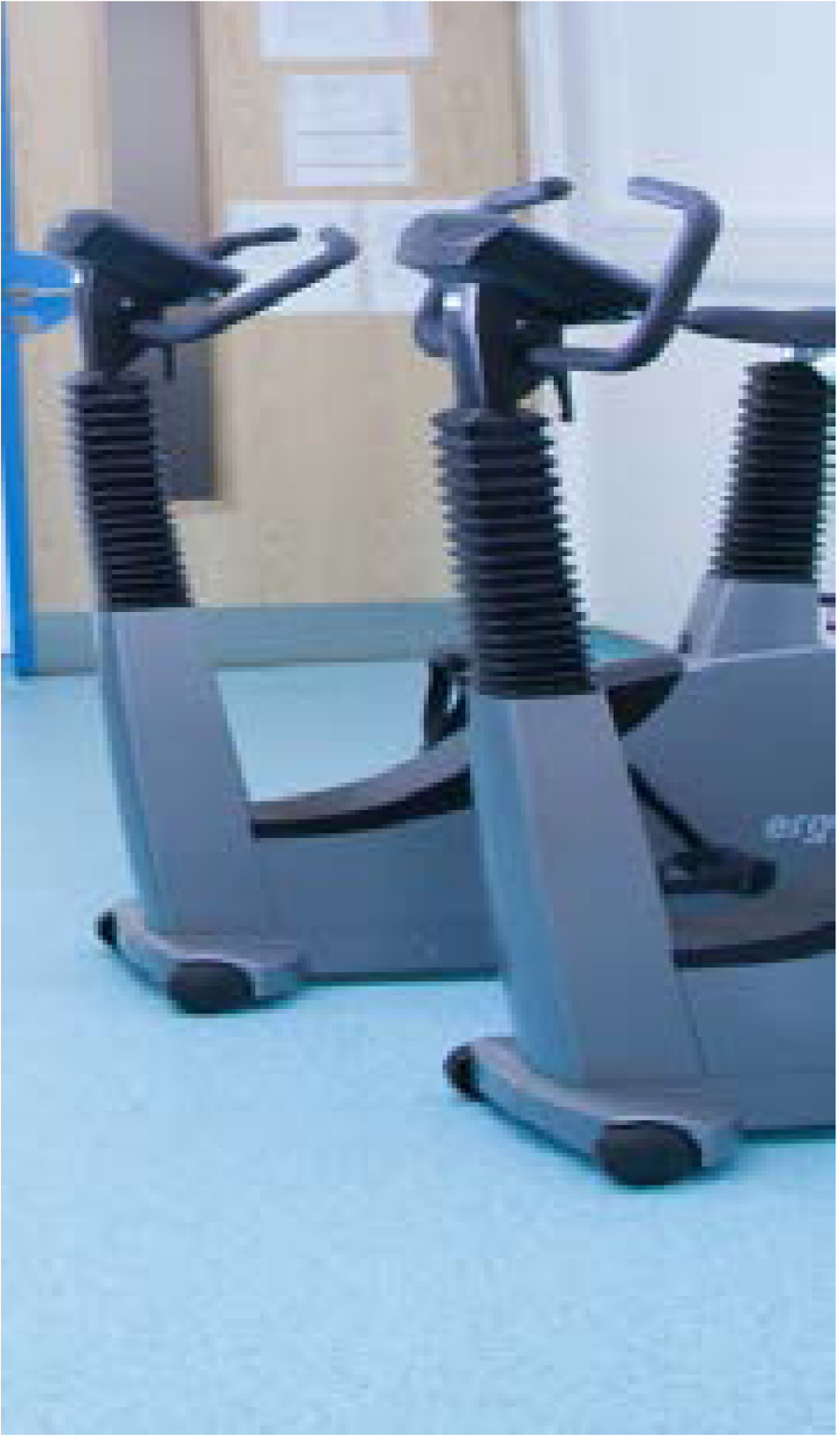
Exercise training cycle ergometers (Optibike Ergoselect 200; Ergoline, GmbH, Germany)

**Fig. 3:**
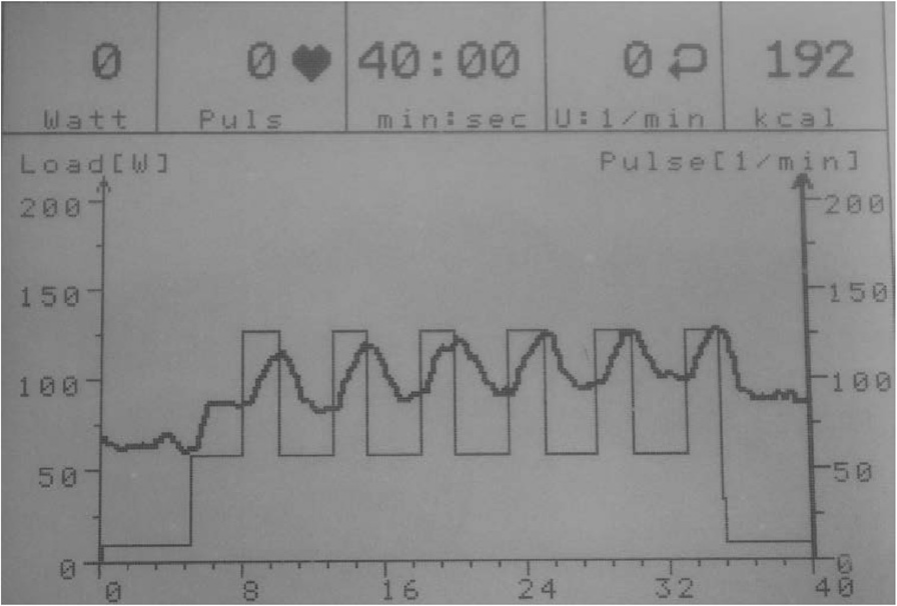
Screen shot of the display on the exercise ergometer at the end of a 40-minute exercise session. This shows power (W) and heart rate (beats.min-1) on the y-axis versus time (min) on the x-axis. The square wave pattern on the background is the preloaded exercise training programme. The sinusoidal pattern on the foreground is the variation in heart rate with the different training intensities recorded over time

The exercise training programme is conducted on a computer-controlled, electromagnetically braked, cycle ergometer (Optibike Ergoselect 200; Ergoline, GmbH, Bitz, Germany) (see Fig. 2). Heart rate is continuously recorded from the R-R interval (Polar FT7, Warwick, UK). The training programme is preloaded on to a chip-and-pin card that executes the interval intensities automatically onto the screen displayed on the cycle ergometer (see Fig. 3).

### Outcome measurements

All patients undergo a series of outcome measure assessments throughout the period of the trial: baseline (pre-neoadjuvant CRT), mid-neoadjuvant CRT and at several time points following completion of the neoadjuvant CRT (weeks 0, 3, 6 and 9) prior to surgery (surgery generally takes place between weeks 6 and 9, depending on the hospital). In UHS only, based on the clinical pathway, some patients are treated with neoadjuvant chemotherapy (four cycles) followed by neoadjuvant CRT. In this instance, an additional research visit is scheduled halfway between the completion of chemotherapy and commencing CRT). The outcome measures used in the EMPOWER trial are summarised in Table 1.

**Table 1:** Outcomes and assessment measures used in the EMPOWER trial

Outcomes	Assessment measure	Pre CRT	Mid chemo-CRT	Mid CRT	Week 0	Week 3	Week 6	Week 9	Day 3 post-surgery	Day 5 post-surgery	Day 8 post-surgery	Day 15 post-surgery	6 Weeks post-surgery
Primary endpoint													
Oxygen uptake at lactate threshold	CPET	X	UHS only		X	X	X	X					
Secondary endpoint													
Health-related quality of life	Questionnaires: EORTC QLQ-30, EQ-5D	X	UHS only	X	X	X	X	X					X
	Semi-structured interviews				X			X					
Physical activity	SenseWear accelerometer	X	UHS only	X	X	X	X	X					
Exploratory endpoint													
Post-operative morbidity	POMS								X	X	X	X	
Cancer staging	MR pelvis mrTRG	X						X					
								X					

*Abbreviations*: *CRT* chemoradiotherapy, *CPET* cardiopulmonary exercise test, *UHS* University Hospital Southampton, *POMS* Post- operative morbidity score, *mrTRG* magnetic resonance tumour regression grade, *MR* magnetic resonance

### Primary outcome

#### Physical fitness

CPET is used to assess physical fitness over a series of time points (which vary in each hospital depending on the cancer treatment and time interval between CRT and surgery): baseline (pre-neoadjuvant CRT); mid chemotherapy-CRT (in UHS only); and post-neoadjuvant CRT (week 0), week 3, week 6 and week 9. All CPETs are performed in the hospital by trained and experienced staff. Every effort is made to coordinate research visits with other clinical appointments. Each CPET is conducted at a similar time of day. Participants are asked to refrain from caffeine and strenuous exercise prior to the test. All CPETs are performed using an electromagnetically-braked cycle ergometer (Ergoline 2000), a 12-lead ECG, non-invasive blood pressure measurement and pulse oximetry, and a metabolic cart (Geratherm Respiratory GmbH, Love Medical Ltd). The incremental rise in work rate is pre-determined using the equation derived by Wasserman and colleagues [22] in which the same work-rate protocol is used for each CPET. This is done in an objective manner aiming for a test duration between 8 and 12 minutes. The ramp protocol equation is as follows:

$$ \begin{array}{c}\hfill \overset{\cdotp }{\mathrm{V}}{\mathrm{o}}_2\kern0.24em \mathrm{unloaded}\kern0.24em \left(\mathrm{ml}.mi{n}^{-1}\right)=150+\left(6\times \mathrm{weight}\kern0.24em \left(\mathrm{kg}\right)\kern0.24em \right)\hfill \\ {}\hfill \overset{\cdotp }{\mathrm{V}}{\mathrm{o}}_2\kern0.24em \mathrm{at}\kern0.24em \mathrm{Peak}\kern0.24em \left(\mathrm{ml}.mi{n}^{-1}\right)\kern0.24em \mathrm{Men}=\left[\mathrm{height}\kern0.24em \left(\mathrm{cm}\right)-\mathrm{age}\left(\mathrm{y}\right)\right]\times 20\hfill \\ {}\hfill \overset{\cdotp }{\mathrm{V}}{\mathrm{o}}_2\; at\kern0.24em \mathrm{Peak}\left(\mathrm{ml}.mi{n}^{-1}\right)\kern0.24em \mathrm{Women}=\left[\mathrm{height}\kern0.24em \left(\mathrm{cm}\right)-\mathrm{age}\left(\mathrm{y}\right)\right]\times 14\hfill \\ {}\hfill \mathrm{Work}\kern0.24em \mathrm{Rate}\kern0.24em \mathrm{increment}\kern0.24em \left(\mathrm{W}.mi{n}^{-1}\right)=\mathrm{Peak}\kern0.24em \overset{\cdotp }{\mathrm{V}}{\mathrm{o}}_2-\left.\overset{\cdotp }{\mathrm{V}}{\mathrm{o}}_2\kern0.24em \mathrm{Unloaded}\right)/100\hfill \end{array} $$

Each participant receive instructions on how to rate the Borg Scale of perceived exertion (Scale 0 to 10), which is a subjective rating of breathlessness and for leg fatigue (assessed every 2 minutes during the test). Additionally, blood pressure is recorded every 2 minutes during the test. Saddle height is measured and recorded at the first CPET and remains constant for all other CPETs. Once the patient is comfortable on the bike, the mask is fitted. The patients are asked to perform an incremental ramp test to the limit of tolerance and to maintain a cycling cadence at 55–65 revolutions per minute (rpm) throughout the test. CPET allows for the derivation of lactate threshold using the modified V-Slope method [23, 24]. The modified V-Slope method identifies the lactate threshold as the tangential breakpoint in the rate of change of ${\overset{\cdotp }{V}}_{C{O}_2}$ relative to $\overset{\cdotp }{\mathrm{V}}{\mathrm{o}}_2$ (oxygen uptake – carbon dioxide output) from the line of unity (‘line of one’) during the incremental stage of the exercise test. The V-slope methods depend solely on the physicochemical reaction of hydrogen ions with bicarbonate, and as such, the occurrence of the breakpoint is independent of the chemoreceptor sensitivity and the ventilatory response to exercise [23]. The inter-observer variability for experienced clinicians is very acceptable [25]. Two independent physiological data assessors are blind to group allocation and are independent to the intervention. The multi-disciplinary team caring for the patients are not provided with any information regarding outcome measures.

### Secondary outcome

#### Health-related quality of life (HRQoL)

HRQoL is assessed in two ways: 1) Semi-structured interviews are conducted at week 0 (following neoadjuvant CRT) and at weeks 6 to 9 (prior to surgery) to explore the patients’ perceptions of quality of life, which allows the patients to focus on personal accounts of their HRQoL within a specific context (for example, exercise participation and active cancer treatment). 2) HRQoL questionnaires are administered over several time points during the study: baseline (pre-neoadjuvant CRT), mid-chemotherapy CRT (in UHS only), mid-neoadjuvant CRT, post-neoadjuvant CRT (week 0), week 3, week 6, week 9 and 30 days post-surgery. The two questionnaires administered at these time points are the EORTC QLQ-30 and EQ-5D. EORTC QLQ-30 is specific to cancer patients and is validated to assess generic aspects of HRQoL. EQ-5D is a simple descriptive profile and a single index value for health status.

### Exploratory outcome measure

#### Physical activity

Daily physical activity is measured over three consecutive days and nights at a series of time points: baseline (pre-neoadjuvant CRT), mid-chemotherapy CRT (in UHS only), post-neoadjuvant CRT (week 0), week 3, week 6 and week 9. Physical activity is measured using a multi-sensory accelerometer ((SenseWear Pro® armband (Model MF-SW, display model DD100); BodyMedia, Inc., Pittsburgh, PA, USA)), which records daily movements, including total energy expenditure, physical activity duration, lying-down time, active energy expenditure, number of steps, sleep duration, total energy expenditure, degree of physical activity, average metabolic equivalent threshold (MET), duration of sensor on body, sleep efficiency, and physical activity level (PAL, which is the ratio of estimated daily energy expenditure to estimated basal metabolic rate).

#### Post-operative morbidity survey (POMS)

Post-operative outcomes will be objectively recorded using POMS on days 3, 5, 8 and 15. The POMS is a validated 18-item tool that addresses nine domains of morbidity relevant to the post-surgical patient: pulmonary, infection, renal, gastrointestinal, cardiovascular, neurological, wound complications, haematological and pain [26, 27]. For each domain, either the presence or absence of morbidity is recorded on the basis of precisely defined clinical criteria.

#### Cancer staging

Cancer down-staging will be assessed using the TNM classification. Rectal cancer staging will be assessed using an abdominal-pelvic CT scan and pelvic MRI scan pre-neoadjuvant CRT and at 9 weeks following completion of the neoadjuvant CRT. The TNM classification of malignant tumours is a cancer staging notation system that gives codes to describe the stage of a solid tumour [28].

### Safety

All adverse events are recorded in the relevant case report form. Adverse events during CPET are reported to the chief investigator, and adverse effects during exercise training (pain and muscle soreness) are recorded in the relevant case report form by the research physiologist/nurse. Fatal or life-threatening serious adverse events (SAEs) are reported within 24 hours of the local site becoming aware of the event. The SAE form documents the nature of the event, date of onset, severity, corrective therapies given, outcome and causality (that is, unrelated, unlikely, possibly, probably, or definitely). Questions concerning adverse event reporting are directed to the chief investigator in the first instance.

### Data analysis

#### Sample size calculation

A sample of 46 patients is required to detect a difference between groups of 2.0 mL.kg^-1.^min^−1^$\overset{\cdotp }{\mathrm{V}}{\mathrm{o}}_2$ at ${\widehat{\uptheta}}_{\mathrm{L}}$ using a two-sample t-test at the 5 % significance level with 90 % power. This is based on a standard deviation of the change in $\overset{\cdotp }{\mathrm{V}}{\mathrm{o}}_2$ at ${\widehat{\uptheta}}_{\mathrm{L}}$ values of 1.8 ml.kg^−1·^min^−1^ and is inflated to allow for 20 % patient drop-out [29]. This estimate was calculated using the sampsi function in Stata/IC 12.0.

#### Procedures for data checking and entering

Data will be double data entered, and data validation will take place according to the procedures set out in the data management plan and data validation plan. Prior to any statistical analysis, all variables will be checked for the number of missing values, impossible values and improbable values. Impossible and improbable values will be defined by clinical opinion. Improbable values will also include values that are outside three standard deviations of the mean value. Any questions regarding the data will go back to the data manager. Descriptive statistics will be calculated for all variables, and distributional assumptions will be checked.

#### Statistical analyses

Descriptive analyses will be carried out to summarise patient characteristics. Continuous variables will be reported as mean (range), mean (SD) or median and inter-quartile range (IQR), depending on distribution, and categorical variables as frequency (%). The effect of neoadjuvant CRT and the exercise intervention on physical fitness will be assessed using a two-sample t-test, and a 95 % confidence interval on the mean difference between the two groups will be presented. In order to assess the sensitivity of results to covariates, a further ‘adjusted’ effect will be calculated using linear regression where up to three candidate covariates will be identified [30]. Basic exploratory analysis of physical fitness at each time-point will be undertaken to investigate the longitudinal response of fitness. Linear mixed modelling will then be employed to compare more formally the fitness at each time-point. Confirmatory analysis using ordinal regression will be used to assess the suitability of the t-test for comparing the HRQoL between the groups. The effect of an in-hospital exercise training programme on physical activity will be analysed in the same way as physical fitness. The presence/absence of post-operative morbidity as defined by a POMS score > 0 will be compared between groups by calculating the relative risk with a 95 % confidence interval. Cancer staging will be reported as tumour, node metastasis version 5 (TNM) staging, Response Evaluation Criteria in Solid Tumours (RECIST) and MR tumour regression grading (mrTRG) criteria. Pathological outcomes will be graded according to the pathological tumour regression grading (TRG) and TNM. Univariate logistic regression analysis will be used to analyse the association between demographic variables (patient age and sex), MRI parameters and pathologic tumour response. This will enable the calculation of odds ratios (OR) for the probability of an unfavourable pathological outcome. Analyses of sensitivity, specificity, positive and negative likelihood ratios will be performed.

The Standard Protocol Items-Recommendations for Interventional Trials (SPIRIT) table provides an overview of the study conduct, review, reporting and interpretation and is presented in Table 2. The final report will follow the Consolidated Standards of Reporting Trials (CONSORT), as well as the Template for Intervention Description and Replication (TIDieR).

**Table 2:** Standard Protocol Items: Recommendations for Interventional Trials (SPIRIT) Table

Administrative details	
Title	A multi-centre, parallel group randomised controlled trial, in locally advanced rectal cancer patients investigating the effects of neoadjuvant chemoradiotherapy and an in-hospital exercise training programme on physical fitness and quality of life in locally advanced rectal cancer patients (The EMPOWER Trial).
Trial registration	ClinicalTrials.gov: NCT01914068 (received: 7 June 2013)
Protocol version	16/01/2015, version 4
Funding	National Institute for Health Research (NIHR) for Patient Benefit Programme (PB-PG-0711-25093).
Roles and responsibilities	MPWG, SJ, MAW, GJK and HBR conceived the study. LL, MAW, GJK, HBR, SMB, TC, PC, CB, DHP, MGM, MPWG and SJ contributed to the study design. LL drafted the manuscript, which underwent revision by all other authors. All authors read and approved the final manuscript.
Sponsor contact information	Dr Mikayala King, Research & Development Department, University Hospital Southampton NHS Foundation Trust.
	Email: Mikayla.King@uhs.nhs.uk
Sponsor and funder	The sponsor and funding course had no role in the design of the study and will not have any role during its execution, analyses, interpretation of the data or decision to submit results.
Committees	The chief investigator (CI: Dr Jack) has overall conduct of the study. The study co-ordinator (SC: Ms Loughney) ensures day-to-day management of the study while working in close contact with the LA Prof. Grocott. The SC is the first point of contact for enquiries from all hospital sites. The SC and CI ensure milestones are achieved in a timely manner.
	The study management group (SMG) is constituted by the CI and all co-investigators. This group is responsible for the strategic management of the study. The SMG maintain quarterly teleconferences and meet face-to-face twice per year (minimum of six meetings). The SMG is chaired by the CI. Local study management groups (LSMGs) comprise participating local investigators, data manager, specialist nurses, clinicians and the local patient representative. Each LSMG is chaired by the local study lead. The LSMGs meet monthly, and the CI will call additional ad hoc meetings. Each hospital has its own ICH-GCP standard with random audit of documents.
Introduction	
Background and rationale	The standard treatment pathway for locally advanced rectal cancer is neoadjuvant chemoradiotherapy (CRT) followed by surgery. Neoadjuvant CRT has been shown to decrease physical fitness, and this decrease is associated with increased post-operative morbidity. Exercise training can stimulate skeletal muscle adaptations such as increased mitochondrial content and improved oxygen uptake capacity, both of which contribute to physical fitness. The aims of the EMPOWER trial are to assess the effects of neoadjuvant CRT and an in-hospital exercise training programme on physical fitness, health-related quality of life (HRQoL), and activity levels, as well as post-operative morbidity and cancer staging.
Comparators	The usual care control group (usual care – no formal exercise training) receive routine care throughout their cancer pathway from diagnosis to surgical resection. No specific advice about exercise training is offered.
Objectives	The aims of this study were to evaluate the following hypotheses:
	Primary hypothesis: A 9-week in-hospital exercise training programme compared with a usual care control group (usual care - no formal exercise training) will result in a clinically significant difference in oxygen uptake at lactate threshold ($\overset{\cdotp }{\mathrm{V}}{\mathrm{o}}_2$ at ${\widehat{\uptheta}}_{\mathrm{L}}$ ; 2.0 mL.kg^-1.^min^−1^) assessed using CPET, following neoadjuvant CRT prior to elective cancer surgery.
	Secondary hypothesis: a) An in-hospital exercise training programme compared with a usual care control group (usual care - no formal exercise training) will result in an improvement in psychological health benefits assessed using semi-structured interviews and questionnaires such as EORTC QLQ-30 and EQ-5D, following neoadjuvant CRT prior to cancer surgery. b) Neoadjuvant cancer treatment will result in a reduction in $\overset{\cdotp }{\mathrm{V}}{\mathrm{o}}_2$ at ${\widehat{\uptheta}}_{\mathrm{L}}$ assessed using CPET.
	Exploratory hypotheses: A 9-week in-hospital exercise training programme compared with a usual care control group (usual care - no formal exercise training) will be associated with 1) a change in physical activity, assessed using SenseWear accelerometer, following neoadjuvant CRT prior to cancer surgery; 2) a change in day 5 surgical morbidity (using POMS) and 3) a change in magnetic resonance (MR)-defined local rectal cancer staging assessed using the TNM classification system (tumour, nodes, metastasis) and tumour regression grading (mrTRG).
Trial design	Parallel group randomised controlled multi-centre trial.
Methods: Participants, interventions, and outcomes	
Study setting	In-hospital. Hospitals include: the University Hospital Southampton NHS Foundation Trust; Aintree University Hospitals NHS Foundation Trust; Royal Hampshire County Hospital, South Tees Hospitals NHS Foundation Trust; and Royal Bournemouth Christchurch Hospitals.
Eligibility criteria	Eligibility criteria for inclusion at cancer diagnosis include the following: age ≥18 years, with MR-defined locally advanced (circumferential resection margin threatened) resectable rectal cancer (≥T2N + M0), undergoing standardised neoadjuvant CRT, and with no distant radiological defined metastasis. Exclusion criteria include the following: inability to give informed consent, non-resectable disease, distant metastasis, inability to perform CPET or bicycle exercise, and patients who declined surgery or neoadjuvant CRT, or who received non-standard neoadjuvant CRT.
Interventions	Patients are randomised (1:1) to either an in-hospital exercise training programme or usual care control group following neoadjuvant CRT (week 0).
	Exercise intervention group
	The exercise training programme was designed to improve physical fitness in the time interval between the end of neoadjuvant CRT and surgery. Exercise training begins on the first week following completion of the neoadjuvant CRT with patients exercising in pairs for camaraderie. The exercise training programme is described below.
Outcomes	Following completion of neoadjuvant CRT (week 0) prior to surgery, patients are randomised to an in-hospital exercise-training programme (aerobic interval training for 6 to 9 weeks) or a usual care control group (usual care -no formal exercise training). The primary endpoint is oxygen uptake at lactate threshold ($\overset{\cdotp }{\mathrm{V}}{\mathrm{o}}_2$ at ${\widehat{\uptheta}}_{\mathrm{L}}$) measured using cardiopulmonary exercise testing assessed over several time points throughout the study. Secondary endpoints include HRQoL, assessed using semi structured interviews and questionnaires, and physical activity levels assessed using activity monitors. Exploratory endpoints include post-operative morbidity, assessed using the Post-Operative Morbidity Survey (POMS), and cancer staging, assessed by using magnetic resonance tumour regression grading.
Participant timeline	CPET is used to assess physical fitness over a series of time points (which vary in each hospital dependent on cancer treatment and time interval between CRT – surgery): baseline (pre-neoadjuvant CRT), mid-chemotherapy-CRT, post-neoadjuvant CRT (week 0), week 3, week 6 and week 9. HRQoL is assessed in two ways: 1) Semi-structured interviews are conducted at week 0 (following neoadjuvant CRT) and at week 6 to 9 (prior to surgery) to explore patients’ perceptions of quality of life, which allows the patients to focus on personal accounts of their HRQoL within a specific context (for example, exercise participation and active cancer treatment); 2) HRQoL questionnaires are administered over several time points during the study: baseline (pre-neoadjuvant CRT), mid-chemotherapy CRT (in UHS only), mid neoadjuvant CRT, post neoadjuvant CRT (week 0), week 3, week 6, week 9 and 30 days post-surgery. Daily physical activity is measured over three consecutive days and nights at a series of time points; baseline (pre neoadjuvant CRT), mid-chemotherapy-CRT (in UHS only), post-neoadjuvant CRT (week 0), week 3, week 6 and week 9. Physical activity is measured using a multi-sensory accelerometer ((SenseWear Pro® armband (Model MF-SW, display model DD100); BodyMedia, Inc., Pittsburgh, PA, USA)) (see Table 1).
Sample size	A sample of 46 patients is required to detect a difference between groups of 2.0 mL.kg^-1.^min^−1^ $\overset{\cdotp }{\mathrm{V}}{\mathrm{o}}_2$ at ${\widehat{\uptheta}}_{\mathrm{L}}$ using a two-sample t-test at the 5 % significance level with 90 % power. This is based on a standard deviation of the change in $\overset{\cdotp }{\mathrm{V}}{\mathrm{o}}_2$ at ${\widehat{\uptheta}}_{\mathrm{L}}$ values of 1.8 ml.kg^−1^.min^−1^ and is inflated to allow for 20 % patient drop-out. This estimate was calculated using the sampsi function in Stata/IC 12.0.
Recruitment	If recruitment is not achieving the target sample size, additional hospitals will be opened to recruit participants.
Methods: Assignment of interventions	
Allocation:	
Sequence generation	Patients are randomised (1:1) to either an in-hospital exercise training programme or usual care control group using the Trans European Network for patient randomisation in clinical trials system (TENAELA System).
Implementation	Randomisation will be generated by the research nurse/Physiologist at each hospital site.
Blinding	Two independent physiological data assessors are blind to group allocation and are independent to the intervention.
Methods: Data collection, management, and analysis	
Data collection methods	For the primary and secondary outcomes, CPET measures changes in physical fitness. Questionnaires and semi-structured interviews will assess changes in HRQoL and physical activity monitoring measures changes in activity levels. Other outcomes are listed in Table 1: Outcomes and assessment measures used in the EMPOWER trial.
Data management	Data will be double data entered, and data validation will take place according to the procedures set out in the data management plan and data validation plan. Prior to any statistical analysis, all variables will be checked for number of missing values, impossible and improbable values. Impossible and improbable values will be defined by clinical opinion. Improbable values will also include values that are outside three standard deviations of the mean value. Any questions regarding the data will go back to the data manager. Descriptive statistics will be calculated for all variables and distributional assumptions checked.
Statistical methods	The effect of neoadjuvant CRT and the exercise intervention on physical fitness will be assessed using a two-sample t-test, and a 95 % confidence interval on the mean difference between the two groups will be presented. In order to assess the sensitivity of results to covariates, a further ‘adjusted’ effect will be calculated using linear regression where up to three candidate covariates will be identified. Basic exploratory analysis of physical fitness at each time-point will be undertaken to investigate the longitudinal response of fitness. Linear mixed modelling will then be employed to compare more formally the fitness at each time-point. Confirmatory analysis using ordinal regression will be used to assess the suitability of the t-test for comparing HRQoL between groups. The effect of an in-hospital exercise training programme on physical activity will be analysed in the same way as physical fitness. The presence/absence of post-operative morbidity as defined by a POMS score > 0 will be compared between groups by calculating the relative risk with a 95 % confidence interval. Cancer staging will be reported as tumour, node metastasis version 5 (TNM) staging, Response Evaluation Criteria in Solid Tumours (RECIST) and MR tumour regression grading (mrTRG) criteria. Pathological outcomes will be graded according to the pathological tumour regression grading (TRG) and TNM). Univariate logistic regression analysis will be used to analyse the association between demographic variables (patient age and sex), MRI parameters and pathologic tumour response This enabled calculation of odds ratios (OR) for the probability of an unfavourable pathological outcome. Analyses of sensitivity, specificity, positive and negative likelihood ratios will be performed.
Methods: Monitoring	
Data monitoring	Data is monitored after the first complete patient at each site to ensure high quality data. Additionally, quality checks are carried out on all CPET’s. An interim analysis will be conducted at the half-way point of the trial.
Harms	All adverse events are recorded in the relevant case report form; adverse events during CPET are reported to the chief investigator and adverse effects during exercise training (pain and muscle soreness) are recorded in the relevant case report form by the research physiologist/nurse. Fatal or life-threatening serious adverse events (SAEs) are reported within 24 hours of the local site becoming aware of the event. The SAE form documents the nature of the event, date of onset, severity, corrective therapies given, outcome and causality (that is, unrelated, unlikely, possibly, probably, definitely). Any questions concerning adverse event reporting are directed to the chief investigator in the first instance.
Ethics and dissemination	
Research ethics approval	Ethical approval: North West Centre for Research Ethics Committees (13/NW/0259, Date: May 2013)
Protocol amendments	Protocol amendments will be agreed and approved by the North West Centre for Research Ethics Committees.
Consent or assent	All potentially eligible patients are identified at multi-disciplinary meetings and approached with written information about the trial at the oncology/surgical outpatient appointment. Patients are contacted by telephone to provide additional information about the trial and to confirm their eligibility. If the patient chooses to participate in this trial, the first research visit is organised where written informed consent is obtained and all baseline measurements are undertaken.
Confidentiality	Data will be entered with all direct patient identifiers removed, patients will be identified by study codes. All physiological data are held in an encrypted format. All data will be stored in a secured locked room.
Declaration of interests	The authors declare that they have no competing interests.
Access to data	Only members of the clinical and research team will have access to patient records.
Dissemination policy	Dissemination of findings to the academic community will be led by the lead applicant guided by the SMG. Dissemination of research findings to patients and the general public will be led by the patient representatives guided by the SMG. The SMG will give public engagement interviews/talks in support of the patient representatives. Active patient involvement will be sought for radio and television interviews, together with issuing of patient statements that can be used at local hospital level to raise awareness regarding prehabilitation and an active life-style in general. The SMG have extensive experience of disseminating medical information to the public and the media, due to the involvement of Professor Grocott as founder member of the Improving Surgical Outcomes Group. He is also involved in the Caudwell Xtreme Everest project, which was widely publicised. The group delivered more than 100 lectures to schools, colleges and in other public forums.

## Discussion

The cancer treatment pathway for locally advanced rectal cancer carries some risk. Neoadjuvant CRT and surgery are associated with side effects and post-operative complications [1, 31]. The level of risk associated with surgery has been examined in a recent European Surgical Outcome Study [32], which reported a mortality rate (3.6 %) that was substantially higher than expected. In the United Kingdom, interest is growing in the use of CPET to objectively risk stratify fitness prior to major surgery in order to better inform a discussion of risk within the collaborative decision making process and to aid personalised perioperative patient care [33]. Physical fitness is an important element of post-operative outcome, and exercise training has the ability to improve clinically important CPET variables [34]. The EMPOWER trial will be the first RCT to investigate the effects of neoadjuvant CRT and an in-hospital exercise training programme on physical fitness, HRQoL and physical activity, as well as post-operative morbidity and cancer staging.

Pre-operative physical fitness is associated with post-operative outcome, with the less fit patients having a greater prevalence of adverse outcomes. Neoadjuvant CRT decreases physical fitness in this patient group, and this decrease is associated with increased post-operative complications [10]. The EMPOWER trial will evaluate the effect of the intervention on physical fitness, HRQoL, physical activity and positive behavioural responses to exercise in the short term. Additionally, the EMPOWER intervention has the potential to effect a long-term benefit because an improvement in perioperative fitness might translate into better post-operative outcomes (longer survival and fewer post-operative complications), cancer down-staging and less demand on hospital resources (reduction in patient length of stay, reduced demand for post-operative high dependency care and associated health economic benefits). The EMPOWER trial will also yield an effect size which can be used to power a further definitive randomised controlled trial and to investigate the association of change in pre-operative physical fitness with exercise and post-operative complications.

Strengths of the EMPOWER trial include the study design with parallel group 1:1 randomisation; the two independent physiological data assessors who are blind to group allocation and independent of the intervention; and that the multi-disciplinary team caring for the patients are not provided with any information regarding predictive measures (for example, CPET variables) ensuring a low risk of confounding by indication [35]. Also, patients are recruited consecutively, all of whom are scheduled for a pre-defined CRT regime and have a homogenous MR-defined rectal cancer staging. Furthermore, the statisticians conducting the analyses are blind to the group allocation until after the analysis is complete. Data are handled using a double entry data system, and impossible or improbable values are questioned. The novelty of the in-hospital exercise training programme in this patient cohort and the statistical analyses used are also worth highlighting.

## Conclusion

In summary, the EMPOWER trial will investigate changes in objectively measured physical fitness and HRQoL with a structured pre-operative exercise training programme. Furthermore, it will also explore changes in physical activity with exercise and any impact on post-operative outcome measures. Additionally, it may inform the development of generalisable exercise programmes for major surgical patients who are identified as high risk (lower physical fitness levels) following cancer treatment.

### Trial status

The trial started patient enrolment in August 2013 and is expected to be completed by the end of December 2015.

## Abbreviations

CPET: cardiopulmonary exercise test
CRT: chemoradiotherapy
HRQoL: health-related quality of life
IQR: inter-quartile range
MET: metabolic equivalent threshold
MR: magnetic resonance
mrTRG: tumour regression grading
NIHR: National Institute for Health Research
PAL: physical activity level
POMS: post-operative morbidity survey
SAE: serious adverse events
TNM: Tumour, Nodes, Metastasis
UHS: University Hospital Southampton
$\overset{\cdotp }{\mathrm{V}}{\mathrm{o}}_2$ at ${\widehat{\uptheta}}_{\mathrm{L}}$: oxygen uptake at lactate threshold
